# Minimally invasive pancreaticoduodenectomy for circumportal pancreas: literature review and report of two type IIIA cases

**DOI:** 10.1186/s40792-024-01979-7

**Published:** 2024-07-29

**Authors:** Hajime Imamura, Tomohiko Adachi, Mampei Yamashita, Ayaka Kinoshita, Takashi Hamada, Hajime Matsushima, Takanobu Hara, Akihiko Soyama, Kazuma Kobayashi, Kengo Kanetaka, Susumu Eguchi

**Affiliations:** Department of Surgery, Nagasaki University Graduate School of Biomedical Sciences, Nagasaki University Hospital, 1-7-1 Sakamoto, Nagasaki, 852-8501 Japan

**Keywords:** Circumportal pancreas, Portal annular pancreas, Periportal annular pancreas, Pancreaticoduodenectomy, Laparoscopic surgery, Robotic surgery, Minimally invasive pancreatectomy

## Abstract

**Background:**

Circumportal pancreas is a rare morphological variant with clinical significance due to the high risk of postoperative pancreatic fistula in patients undergoing pancreaticoduodenectomy. Type IIIA (suprasplenic anteportal) is the most common type of circumportal pancreas. We present two cases of type IIIA treated with minimally invasive pancreaticoduodenectomy, and review the literature on patients with circumportal pancreas who underwent pancreatic surgery.

**Case presentation:**

*Case 1*: Laparoscopic Pancreaticoduodenectomy for Non-functioning Pancreatic Neuroendocrine Neoplasm with Circumportal Pancreas. A 69-year-old female with no prior medical history presented with a pancreatic head mass detected during routine ultrasound. CT revealed a 20 mm hypervascular tumor in pancreas head and a suprasplenic circumportal pancreas with an anteportal duct. The main pancreatic duct (MPD) was not in the parenchyma on the dorsal side of the portal vein (PV). Laparoscopic pancreaticoduodenectomy was performed. The anteportal side was resected with an ultrasonic device, and the retroportal side with a mesh-reinforced stapler. Pancreaticojejunostomy was performed without complications. *Case 2*: Robot-assisted Pancreaticoduodenectomy for Pancreatic Head Cancer and Non-functioning Pancreatic Neuroendocrine Neoplasm in the pancreatic tail with Circumportal Pancreas. A 72-year-old male with no prior medical history presented with a dilated main pancreatic duct on ultrasound. Diagnosed with pancreatic head cancer (Stage IIA), he underwent neoadjuvant chemotherapy. Contrast-enhanced CT revealed pancreatic cancer in the head and a tumor in the tail with unknown pathology. Robot-assisted pancreaticoduodenectomy was performed, and pancreatectomy on the left side of the tail tumor was planned. Intraoperative findings revealed a circumportal pancreas with the MPD not running through the dorsal parenchyma. After resected the parenchyma on the left side of the tail tumor, parenchyma on the dorsal side of the PV was dissected using SynchroSeal®. Pancreaticojejunostomy was performed without complications. The postoperative course was uneventful.

**Conclusions:**

The optimal location and method of pancreatic resection should be selected according to the type of circumportal pancreas and the location of the lesion to be resected to minimize the risk of pancreatic fistula. Minimally invasive surgery for circumportal pancreas remains challenging even for surgical teams with sufficient experience and skills, and careful consideration are necessary for its application.

## Introduction

Various congenital or morphological abnormalities can occur during embryonic development of the pancreas, including pancreas divisum [1], annular pancreas [2], and agenesis of the dorsal pancreas [3]. Circumportal pancreas (CP) is a rare morphological variant of the pancreas, where the pancreatic parenchyma from the uncinate process fuses with the body of the pancreas, resulting in anomalous encasement of the portal vein (PV) and/or superior mesenteric vein (SMV) by an annulus of pancreatic tissue [4]. Based on the relationship between the fusion of the uncinate process and the body of the pancreas (i.e., the annulus) with respect to the splenic vein, CP is subdivided into suprasplenic (type A), infrasplenic (type B), or mixed (type C) [5]. CP has also been classified by Joseph et al. into three types: type I, fusion of the ventral bud of the pancreas with the body and retroportal main pancreatic duct (MPD); type II, associated with pancreas divisum; and type III, PV encasement by the uncinate process with a normal anteportal MPD [6].

Determining the basic anatomy of the pancreas is of utmost importance to surgeons involved in pancreatic surgery. Based on this knowledge, appropriate intraoperative manipulation and judgment are required for rare congenital anomalies incidentally encountered during pancreatic surgery. Although CP is very rare, surgical treatment of CP requires sufficient anatomical knowledge as well as surgical strategies appropriate to the type of CP. To date, previous reports based on open surgery have suggested extended resection [7] or a standard plane of resection with suturing of the retroportal portion [8]. However, reports on the usefulness, safety, and surgical techniques of minimally invasive pancreatectomy (MIP) for CP are limited.

We performed laparoscopic and robotic pancreaticoduodenectomy for two cases of CP type IIIA, as classified by Joseph-Karasaki et al. Therefore, we report these cases with a review of the literature on type IIIA and discuss the anatomical aspects of CP and the key points of the surgical technique and strategy for MIP.

## Materials and methods

A literature search was conducted using the data available from PubMed Central (https://www.ncbi.nlm.nih.gov/pmc/), between January 2008 and March 2024. All articles published in English were searched using the terms “circumportal pancreas,” “periportal pancreas,” and “portal annular pancreas.” All articles describing CP with surgical resection of the pancreas or pancreatic surgery were included in the literature review. After excluding ineligible articles from the title and abstract, they were evaluated based on the description in the article regarding patient information, primary tumor, surgical approach, management of stump of the dorsal side pancreas, anastomotic method (pancreaticogastrostomy or pancreaticojejunostomy), and data regarding postoperative pancreatic fistula (POPF) and type IIIa cirumportal pancreas with pancreaticoduodenectomy. In addition to the cases reported in the literature, cases of CP experienced in our department were included in the data and presented as a case report.

Informed consent was obtained from the patient for publication of this article. The authors received and archived patient consent for intraoperative video or picture recording/publication prior to the video recording of the procedure. The study protocol was approved by the Ethics Committee of Nagasaki University Hospital. This study was conducted in accordance with the Declaration of Helsinki and ethical guidelines for clinical studies of the Ministry of Health, Labor, and Welfare of Japan.

## Case presentation

*Case 1:* The patient was a 69-year-old female. No medical history, including abdominal surgery. The patient’s medical history included an abdominal ultrasound during a physical examination that revealed a mass in the pancreatic head. On examination, she was diagnosed with non-functioning pancreatic neuroendocrine neoplasms and referred to our department for surgery.

**Fig. 1:**
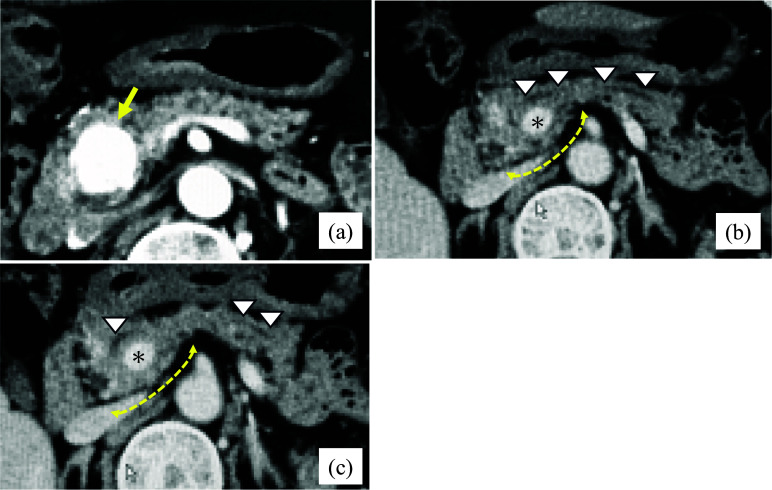
Preoperative contrast-enhanced CT; Case 1. **a** A 20 mm hypervascular tumor is detected in the head of the pancreas (yellow arrow). **b** Suprasplenic CP with anteportal MPD. **c** The pancreatic duct does not run in the pancreatic parenchyma on the dorsal side of the PV. Dotted yellow line: range of the dorsal parenchyma. Asterisks indicate the PV. White triangle: MPD

Contrast-enhanced computed tomography, a 20 mm hypervascular tumor in the head of the pancreas (Fig. 1a). In addition, a suprasplenic CP with an anteportal duct was detected (Fig. 1b, c). The MPD is not located in the parenchyma on the dorsal side of the PV. Laparoscopic pancreaticoduodenectomy was planned for the preoperative diagnosis of non-functioning pancreatic neuroendocrine neoplasms in the pancreatic head with CP. Regarding the planned location of the pancreatic resection, the anteportal side was resected with an ultrasonic coagulation cutting device, whereas the retroportal side was resected with a mesh-reinforced stapler.

**Fig. 2:**
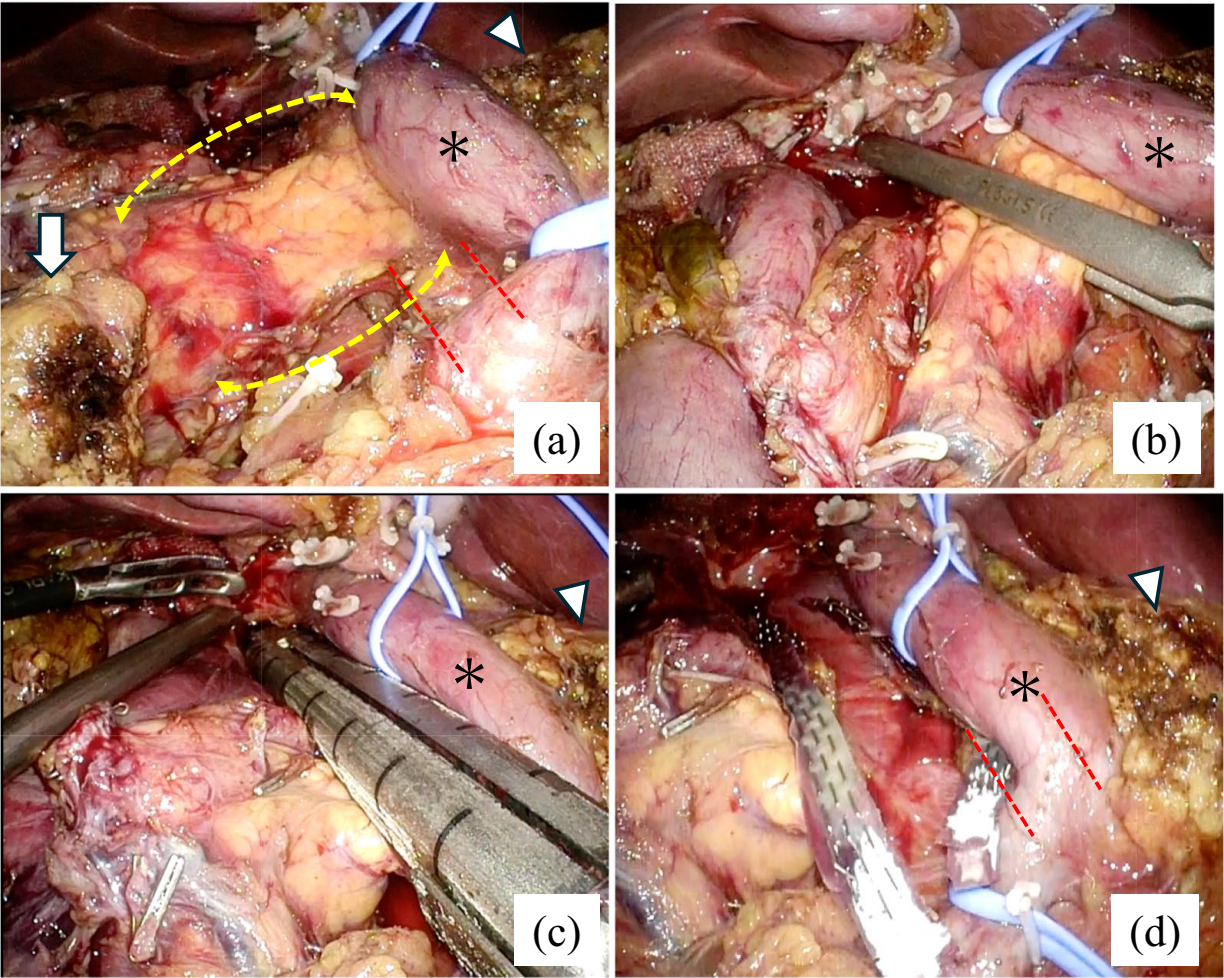
Intraoperative findings of laparoscopic pancreaticoduodenectomy. **a** After resecting the anteportal side of the pancreas, the dorsal side of the CP is exposed. **b** Precompression of the dorsal side of the CP parenchyma using an intestinal clip. **c** The dorsal side of the CP parenchyma dissection by mesh-reinforced stapler. **d** Visual appearance after pancreas resection. Dotted yellow line: dorsal side of the circumportal pancreatic parenchyma. The asterisk: the PV. White triangle: stump of the remnant pancreas. Arrow: Specimen stump, Dotted red line: the outline of the superior mesenteric artery

**Fig. 3:**
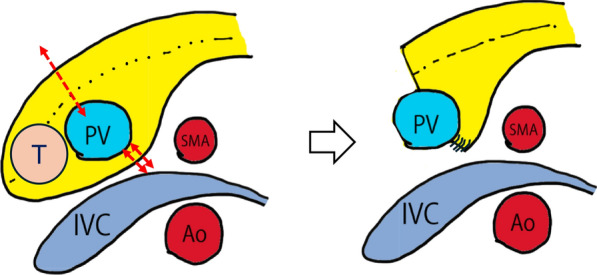
Shown in the axial view: the tumor was located in the head of the pancreas. The ventral side of the pancreas was transected directly above the portal vein. The dorsal side was transected using a stapler (Indicated by double red dashed lines). T: NEN, PV: Portal vein, SMA: Superior mesenteric artery, IVC: Inferior vena cava, Ao: Aorta, Dotted line: MPD

Intraoperative findings showed that the pancreatic parenchyma was connected to the remnant pancreas through the dorsal side of the PV during dissection toward the root of the common hepatic artery while preserving the plexus of the superior mesenteric artery (SMA) (Fig. 2a). After precompression of the pancreatic parenchyma with an intestinal clip on the right side of the PV (Fig. 2b), the pancreatic parenchyma on the dorsal side of the PV was dissected using a mesh-reinforced stapler (Fig. 2c, d). To avoid damage to the SMA during this procedure, it was crucial to ensure that the right side of the SMA nerve plexus was clearly visible and that the head side of the dorsal pancreas was detached prior to dissection. The schematic diagrams before and after pancreatic transection are shown in Fig. 3.

In the reconstruction, MPD had a single hole, and a pancreaticojejunostomy was performed. In laparoscopic pancreaticoduodenectomy, a 5–7 cm incision is made in the upper abdomen for specimen extraction. Subsequently, a pancreaticojejunostomy is performed through this small incision using a modified Blumgart technique. In this modified Blumgart technique, the pancreatic parenchyma and the lifted jejunum are secured using 4–0 non-absorbable sutures with custom-made straight needles. If the width of the pancreatic parenchyma is within 3 cm, two sutures are placed, with one crossing the main pancreatic duct. If it exceeds 3 cm, three sutures are used, with one crossing the duct in the middle. After suturing, the lifted jejunum is adequately placed on the dorsal side of the pancreas and temporarily tied on the ventral side of the pancreatic parenchyma. For the pancreaticojejunostomy, if a 5 Fr stent can be inserted, six interrupted sutures at 60-degree intervals are used. If only a 4 Fr stent can be placed, four interrupted sutures using 5-0 PDS are employed. After securing the posterior wall again, the lifted jejunum and the ventral wall of the pancreatic parenchyma are sutured using straight needles, and the pancreaticojejunostomy is covered and tied securely. The pancreaticojejunostomy was performed without complications, and the postoperative course was uneventful. The final pathological diagnosis was a non-functioning pancreatic neuroendocrine neoplasm (G1).

*Case 2:* The patient was a 72-year-old male. no medical history, including abdominal surgery. The present medical history revealed that the MPD was dilated on abdominal ultrasonography during medical examination. After detailed examination, he was diagnosed with pancreatic head cancer [cT3N0M0: cStage IIA, classified according to the General Rules for the Study of Pancreatic Cancer (8th edition)]. After neoadjuvant chemotherapy with gemcitabine and S-1, no tumor growth or disease progression was observed, and robot-assisted pancreaticoduodenectomy was performed.

**Fig. 4:**
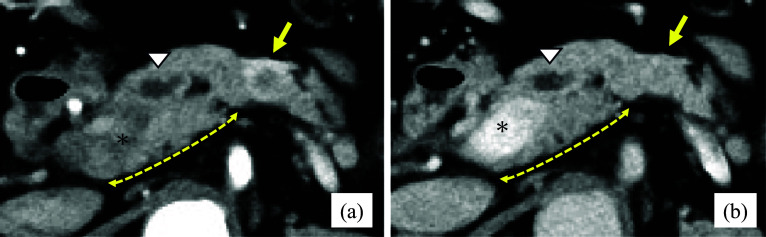
Preoperative contrast-enhanced CT; Case 2. **a** Arterial phase: CP and a tumor with contrast effect in the pancreatic tail (yellow arrow). **b** Portal phase: suprasplenic CP with anteportal MPD. The pancreatic duct did not run through the pancreatic parenchyma on the dorsal side of the PV. Dotted yellow line: range of the dorsal parenchyma. The asterisk: the PV. White triangle: MPD. Yellow arrow: Tumor with a contrast effect

Contrast-enhanced CT detected pancreatic cancer in the pancreatic head, and simultaneously, a tumor with a contrast effect was found in the pancreatic tail (left side of the SMA); however, the pathological diagnosis was unknown (Fig. 4a, b). Therefore, pancreatectomy was planned on the left side of the tumor in the pancreatic tail (extended resection [7]). In addition, a suprasplenic CP with an anteportal duct was detected. The MPD did not run through the parenchyma on the dorsal side of the PV.

**Fig. 5:**
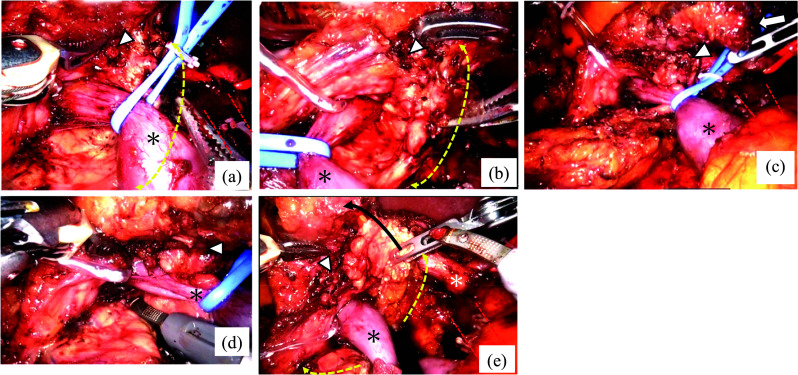
Intraoperative findings of robotic-assisted pancreaticoduodenectomy. **a** The dorsal part of the CP was identified from the right side of the PV to the dorsal side. **b** The area of fusion of the CP from the left side of the PV to the pancreatic body is identified. **c** Overall view after resection of the left side of the tumor in the pancreatic tail. Fusion sites on the ventral and dorsal sides of the circumportal pancreatic parenchyma were also identified. **d** The pancreatic parenchyma is dissected on the right side of the PV. **e** The fourth arm holding the resected dorsal pancreatic stump, which is rolled up towards the patient's right side. Dotted yellow line: range of the dorsal parenchyma. The asterisk: the PV. White triangle: fusion sites on ventral and dorsal sides. Dotted red line: the outline of the superior mesenteric artery. Black line: direction of the roll-up. The white asterisk: common hepatic artery

**Fig. 6:**
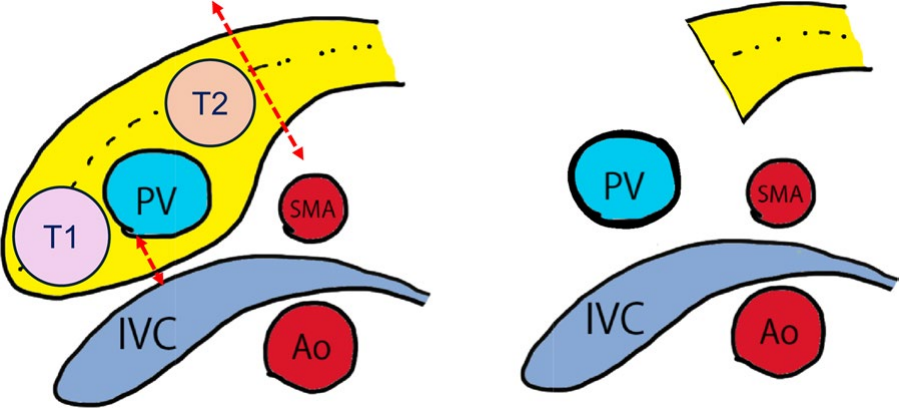
Shown in the axial view: the tumor was located in two places: the head and the body of the pancreas. We resected the pancreatic body on the left side of the tumor(#2) in the pancreatic tail and detached the SMV from the surrounding pancreatic parenchyma. After confirming separation of the SMA and dorsal parenchyma, the pancreatic parenchyma was dissected on the right side of the PV. Upon completing the resection, the pancreatic head and body were rolled to the right side of the patient to avoid damaging the remnant tissue. T1: Pancreatic head cancer, T2: NEN, PV: Portal vein, SMA: Superior mesenteric artery, IVC: Inferior vena cava, Ao: Aorta, Dotted line: MPD

The da Vinci Xi System (Intuitive Surgical Inc., Sunnyvale, CA, USA) was used. Intraoperative findings revealed that the pancreatic parenchyma was connected to the body of the pancreas via the dorsal PV. By moving the blue tape, which was marked at the suprasplenic level of the PV, to the left or right, the pancreatic parenchyma on the retroportal side was well-identified (Fig. 5a, b). To complete the pancreatectomy, we first resected the body of the pancreas on the left side of the tumor in the pancreatic tail and detached the SMV from the surrounding pancreatic parenchyma. After dissection of the parenchyma on the left side of the tumor, fusion sites on the ventral and dorsal sides of the pancreatic parenchyma were identified (Fig. 5c). After confirming that the SMA and dorsal parenchyma were separated, the pancreatic parenchyma was dissected using SynchroSeal® (Intuitive Surgical, Inc., Sunnyvale, CA, USA) on the right side of the portal vein (PV) (Fig. 5d). To prevent damage to the SMA, whenever further separation of the dorsal pancreas from the SMA is required, the dissection should be performed while slightly leaning on the pancreas to preserve the SMA nerve plexus. After the resection was completed, the pancreatic head and body parenchyma were separated from the PV by rolling up to the right side of the patient to avoid damage to the remnant tissue, finally completing the pancreatic head resection (Fig. 5e). The schematic diagrams before and after pancreatic transection are shown in Fig. 6.

In the reconstruction, MPD was identified as a single opening in the pancreatic tail. The robot-assisted pancreaticojejunostomy was performed following the same procedure as described in the modified Blumgart technique in case 1, the details of which have been previously reported [8]. The pancreaticojejunostomy was performed without complications, and the postoperative course was uneventful. The final pathological diagnosis was invasive ductal carcinoma (pStage IIB) in the pancreatic head and neuroendocrine neoplasm (G1) in the pancreatic tail, with negative surgical margins.

## Results of literature review

In a literature review, there were 28 cases of type IIIa CP in which pancreaticoduodenectomy was performed [7, 9–21], including our two cases. Detailed results are presented in Table 1. The median age of the patients was 71(46–84) years, and there were 19 males and 9 females. The primary diseases were pancreatic ductal adenocarcinoma (*n* = 9), ampullary cancer (*n* = 5), bile duct cancer (*n* = 5), duodenal cancer (*n* = 3), intraductal papillary mucinous neoplasm (*n* = 3), neuroendocrine neoplasm (*n* = 2) (including duplicate #15 in Table 1), and others (*n* = 3). The surgical approach was open in 25 cases, laparoscopic in two, and robotic in one. Regarding the management of the stump on the dorsal side of the pancreas, the number of management methods was as follows: stapled (*n* = 11), extended resection (*n* = 7), interrupted sutures (*n* = 3), cautery (*n* = 2), stump into pancreatogastrectomy (*n* = 2), ligated (*n* = 1), not described (*n* = 2). Regarding the presence of POPF, none was found in total 15 cases, details as follows; Grade A: 2 cases, Grade B: 9 cases, Grade C: 1 case, and not described in 1 case.

**Table 1:** Surgical cases for circumportal pancreas in type IIIA

No.	year	Author	Ref. #	Age	Gender	Primary tumor	Approach	Retroportal side of pancreas*	Anastmosis	POPF Grade
1	2011	Ishigami K, et al	10	65	M	PDAC	Open	ND	ND	ND
2	2012	Jang JY, et al	11	71	M	IPMN	Open	Stapled	PJ	B
3	2012	Shonaka T, et al	12	53	M	PDAC	Open	Both stumps-PG	PG	A
4	2013	Kobayashi S, et al	13	61	F	Ampullary	Open	Interrupted sutures	PJ	No
5	2016	Matsumoto I, et al	14	78	M	Duodenal	Open	Both stumps-PG	PG	No
6	2016	Pardiwala KH, et al	15	81	F	Duodenal	Open	Ligated	PJ	No
7	2017	Balila RM, et al	16	72	M	Duodenal gastrointestinal stromal tumor	Open	ND	PJ	A
8	2017	Kulemann B, et al	7	67	M	PDAC	Open	Extended resection	PJ	No
9	2017	Luu AM, et al	17	73	F	PDAC	Open	Extended resection	PJ	No
				81	M	Ampullary	Open	Extended resection	PJ	No
10	2017	Ohtsuka T, et al	18	46	M	Bile duct	Open	Stapled	ND	B
				64	M	IPMN	Open	Extended resection	ND	B
				66	M	PDAC	Open	Stapled	ND	No
				76	F	Bile duct	Open	Stapled	ND	No
				77	F	PDAC	Open	Extended resection	ND	No
				84	F	Bile duct	Open	Extended resection	ND	B
11	2018	Dhanapal B, et al.	19	47	M	Ampullary	Open	Interrupted sutures	PJ	No
12	2018	Kiuchi R, et al.	9	55	M	Ampullary	Open	Cautery	PJ	C
				65	M	IPMN	Open	Stapled	PJ	B
				66	M	Duodenal	Open	Stapled	PJ	B
				74	M	Bile duct	Open	Stapled	PJ	B
				76	M	Bile duct	Open	Stapled	PJ	B
				78	M	PDAC	Open	Cautery	PJ	No
				79	F	Ampullary	Open	Stapled	PJ	No
13	2021	Pandrowala S, et al.	20	58	M	PDAC	Open	Interrupted sutures	PJ	B
14	2022	Nagai K, et al.	21	78	F	Metastatic tumor from renal cell carcinoma	Laparoscopic	Stapled	PJ	No
15	2024	Our case		69	F	Neuroendocrine neoplasm	Laparoscopic	Stapled	PJ	No
				72	M	PDAC and neuroendocrine neoplasm	Robotic	Extended resection	PJ	No

*PDAC* pancreatic ductal adenocarcinoma, *IPMN* intraductal papillary mucinous neoplasm, *PG* pancreaticogastrostomy, *PJ* pancreaticojejunostomy, *POPF* postoperative pancreatic fistula, *ND* not described.*: Management of stump of the retroportal side of pancreas parenchyma

## Discussion

Encircling the PV by the pancreatic parenchyma, which is normal in pigs [22], is an extremely rare and poorly recognized anatomical variant in humans. The first case of CP was reported by Sugiura et al. in 1987 [23]. A recent large study reported an incidence of 0.8% (55/6813 cases), which was determined using thin-section multidetector computed tomography [24]. As CP is asymptomatic, cases of incidental detection during CT [10], intraoperative pancreatic surgery [4, 6], or islet isolation [3, 25] have been reported. As previously mentioned, CP was classified by Karasaki [5] and Joseph [6]. Each focuses on the relationship between the inflow point of the splenic vein and the PV, the fusion position of the pancreatic parenchyma (types A, B, and C), and the running path of the MPD (types I, II, and III). As the two cases presented this report, suprasplenic (type A) CP with anteportal duct (type III) was reported to be the most common variant, with a frequency of type IIIA (44.4–82%), followed by type IA (5–27.8%) [26, 27]. Imaging diagnostics, including the visualization of the MPD course, should involve not only contrast-enhanced CT but also MRCP prior to surgery, as this approach has been shown to reveal a more accurate course of the MPD and its utility has been reported [20]. Specifically, MRCP provides detailed visualization of the ductal course and its relationship to the portal vein. However, it should be noted that the inability to visualize a retroportal ductal structure does not rule out its presence.

This morphologic variant is clinically critical because patients undergoing pancreatic surgery, especially pancreaticoduodenectomy, are at a high risk of developing POPF. A systematic review analyzing 21 previous studies reported a POPF rate of 46.7% in CP cases (12 pancreaticoduodenectomies and three distal pancreatectomies) [27]. A recent review also reported that POPF was present in 42.55% of the patients with clinically relevant POPF (34%) [20]. As a strategy to prevent POPF in CP, it is important to check the course of MPD using preoperative imaging and determine the location and extent of pancreatic resection accordingly. It is also important to simulate the pancreatic resection surface that is formed after resection before surgery. Pancreaticoduodenectomy for CP usually requires an incision not only in the annulus but also in the anteportal pancreatic neck, thus creating two dissecting planes in the pancreas. In contrast, in the mixed vein type (Karasaki type C), there may be up to three pancreatic dissecting planes [5]. The risk of PF increases as the number of dissecting planes increases; ideally, a single dissecting plane would be the best way to reduce the risk of POPF, since the pancreatic duct would also have a single hole. This would require an extended, wide pancreatic parenchymal resection [7, 20], but it has been pointed out as a disadvantage that it leads to unexpected injury to the pancreatic parenchyma and capsule, as the area of the cut surface is usually larger in the pancreatic body than at the level of the PV/SMV [18] and may cause deterioration of the remnant pancreatic function [5, 17, 20]. Although efforts should be made to avoid such disadvantages, as in case 2, it is necessary to consider the location of the resection depending on the underlying disease and the tumor location. Therefore, it is difficult to standardize procedures according to the type of CP.

The results of previous reports on the most common type of IIIA (suprasplenic anteportal type), including our two cases, are summarized in Table 1. The results of this review showed that various techniques are used for resection of the dorsal side of the pancreas, which is an important aspect of the procedure for CP. The resection approach for type IIIA can be divided in two ways: the first is to divide the pancreas on the ventral and dorsal sides of the PV, resulting in two planes; the other is to divide the pancreas on the left side of the parenchymal fusion, resulting in a single plane (extended resection). This may depend on the institution's policy and surgeon's preference. The relationship between POPF and dissection techniques remains controversial, with no clear consensus on which dissection technique is superior in preventing POPF [18, 20]. One of the methods, the dissection procedure using a linear stapler, is simple and easy and is particularly well suited for use in MIP. The stapler method has been reported to have an advantage over extended resection in preserving pancreatic parenchyma and function [18]. Robot-assisted pancreaticoduodenectomy using a linear stapler with a progressive stepwise compression technique has been reported [28]. Resection of the dorsal pancreas using a linear stapler is the preferred choice for type III.

The primary advantage of extended resection is the achievement of a single pancreato-intestinal anastomosis on a single plane of pancreatic resection, which reduces the risk of potential pancreatic leakage [7]. However, it has been noted that extended resection of the CP is technically difficult in MIP [28]. This is because the pancreas must first be dissected at two points, ventral and dorsal to the PV, and the remnant pancreas must then be mobilized to the left side of the SMA/SMV. An additional pancreatectomy must be performed to obtain a single plane of dissection, which complicates the procedure. Although there have been reports of laparoscopic [21] or robot-assisted resection [28] of CP of type III, all were performed with a stapler for resection of the dorsal side of the PV. Our report is the first to describe extended resection performed using a robot-assisted approach. In our approach to robotic surgery, we first dissected the body of the pancreas, detached the SMV from the surrounding pancreatic parenchyma, performed parenchymal dissection on the dorsal side of the PV using an energy device, and proceeded with dissection between the parenchyma and dorsal structures as if rolling the pancreas up to the right side of the patient to avoid damage to the remnant tissue, resulting in specimen release. This could be done safely because the caudal field of view provides a magnified view of the pancreatic parenchyma and dorsal remnant structures. Although extended resection can be a complicated procedure, robot-assisted resection can be performed safely by moving the specimen to the patient's right side and detaching it from residual tissue. However, MIS for CP remains challenging even for surgical teams with sufficient experience and skills, and careful consideration and judgement are necessary for its application, including the handling of the dorsal pancreas.

## Conclusion

In pancreatic surgery for CP, the location and method of pancreatic resection that could reduce the risk of developing a PF should be selected based on the type of CP and the location of the tumor to be resected by examining preoperative images in detail. MIP using the advantages of the caudal view is a useful and safe option for CP resection.

## Data Availability

The data sets used and/or analysed during the current study are available from the corresponding author on reasonable request.

## Abbreviations

CP: Circumportal pancreas
PV: Portal vein
MPD: Main pancreatic duct
MIP: Minimally invasive pancreatectomy
POPF: Postoperative pancreatic fistula
